# Identifying Cancer Driver Genes Using Replication-Incompetent Retroviral Vectors

**DOI:** 10.3390/cancers8110099

**Published:** 2016-10-25

**Authors:** Victor M. Bii, Grant D. Trobridge

**Affiliations:** 1 College of Pharmacy, Washington State University, WSU Spokane PBS 323, P.O. Box 1495, Spokane, WA 99210, USA; victor.bii@wsu.edu; 2 School of Molecular Biosciences, Washington State University, Pullman, WA 99164, USA

**Keywords:** insertional mutagenesis, replication-incompetent retroviral vector, gammaretroviral (γRV) vector, lentiviral (LV) vector, cancer driver genes, drug targets, biomarker

## Abstract

Identifying novel genes that drive tumor metastasis and drug resistance has significant potential to improve patient outcomes. High-throughput sequencing approaches have identified cancer genes, but distinguishing driver genes from passengers remains challenging. Insertional mutagenesis screens using replication-incompetent retroviral vectors have emerged as a powerful tool to identify cancer genes. Unlike replicating retroviruses and transposons, replication-incompetent retroviral vectors lack additional mutagenesis events that can complicate the identification of driver mutations from passenger mutations. They can also be used for almost any human cancer due to the broad tropism of the vectors. Replication-incompetent retroviral vectors have the ability to dysregulate nearby cancer genes via several mechanisms including enhancer-mediated activation of gene promoters. The integrated provirus acts as a unique molecular tag for nearby candidate driver genes which can be rapidly identified using well established methods that utilize next generation sequencing and bioinformatics programs. Recently, retroviral vector screens have been used to efficiently identify candidate driver genes in prostate, breast, liver and pancreatic cancers. Validated driver genes can be potential therapeutic targets and biomarkers. In this review, we describe the emergence of retroviral insertional mutagenesis screens using replication-incompetent retroviral vectors as a novel tool to identify cancer driver genes in different cancer types.

## 1. Introduction

Cancer is currently the second leading cause of death in the U.S. [1]. In the past decades, tremendous advances in screening methods for genes that drive cancer progression have identified previously unrecognized cancer genes and underlying cancer-associated signaling pathways [2,3,4,5]. The identification of cancer genes has significantly improved available therapies for cancer patients. For example, identifying the *BCR-ABL* driver mutation in chronic myeloid leukemia (CML) has led to remarkable patient outcomes with imatinib. Specific targeting of the *BCR-ABL* fusion gene with the kinase inhibitor imatinib increases the five-year patient survival rate to 90% [6]. In breast cancer, overexpression of the human epidermal growth factor receptor 2 (*HER 2*) gene results in a poor prognosis with an increased risk of metastasis and tumor recurrence [7]. The monoclonal antibody trastuzumab that targets HER 2+ breast cancer tumors when used in combination with chemotherapy results in a 33% reduction in the risk of death among HER 2+ patients [8]. These examples show that cancer driver genes can be therapeutic targets for small molecule drugs that can improve patient outcomes. However, despite these advances, identifying driver genes in most cancer types still remains challenging. The high-throughput sequencing approaches that are widely used to identify cancer genes are limited by their inability to efficiently distinguish the driver genes from a wide spectrum of passenger genes [9,10,11]. Therefore, screening approaches that can isolate driver genes from passenger genes are urgently needed.

Insertional mutagenesis techniques using retroviruses and transposons have gained wide application in cancer gene discovery [12,13,14,15,16]. However, both methods have significant limitations. Transposons have been used for germline [17,18] and genome-wide somatic insertional mutagenesis in mice [13,19], but they require transgenic models which can be time consuming to develop [20]. Replicating retroviruses are limited to cells and tissues in which they can efficiently replicate. The use of either replicating retroviruses or transposons for insertional mutagenesis screens is also limited by additional insertional passenger mutations that occur after the driving mutations, and can complicate identification of true driver genes.

Replication-incompetent retroviral vectors have major advantages over both replicating retroviruses and transposons. After the initial insertional mutagenesis event, the integrating replication-incompetent vector does not create additional insertional mutagenesis events in the host cell genome. In addition, replication-incompetent vectors do not need to be able to replicate in a target tissue, they only need to be able to stably infect target cells. Thus, due to the broad tropism of current replication-incompetent vectors mediated by envelope pseudotyping, mutagenesis screens can be performed for essentially any cancer type. To date, replication-incompetent vectors have been used to successfully identify cancer driver genes in breast, prostate, liver and pancreatic cancers [21,22,23,24,25]. Replication-incompetent retroviral vectors integrate into the host genome and dysregulate proto-oncogenes via well-known mutagenic mechanisms including enhancer-mediated activation of nearby gene promoters [26]. An important advantage of using replication-incompetent retroviral vectors is that the level of insertional mutagenesis can be controlled by adjusting the multiplicity of infection (MOI). Taken together, these features have led to the development of replication-incompetent retroviral vectors as powerful tools for insertional mutagenesis screens to identify driver genes in different cancer types.

**Figure 1:**
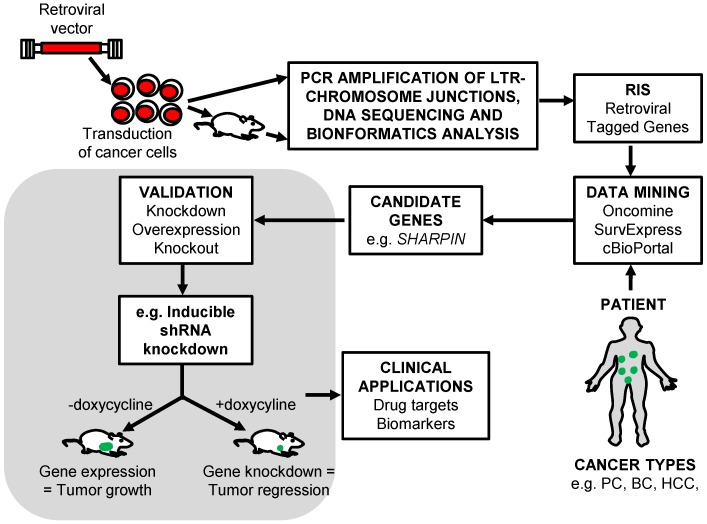
Retroviral insertional mutagenesis screen schematic outline for identification of cancer driver and drug resistance genes. The cancer cells are transduced with retroviral vector, selected and cultured in vitro or xenotransplanted in vivo. The genomic DNA is obtained from tumors or drug resistant clones and the LTR-chromosomal junction determined. High-throughput sequencing and bioinformatics analysis is performed to map the retroviral integration site (RIS) in the genome and identify nearby tagged cancer driver or drug resistant genes. Expression of candidate genes are compared with patient data acquired from publicly available databases and candidate dysregulated gene identified. Candidate driver genes are independently validated to show their involvement in tumor initiation, progression, or drug resistance. PC (prostate cancer), BC (breast cancer) and HCC (hepatocellular carcinoma).

In this review, we describe the use of replication-incompetent retroviral insertional mutagenesis screens to effectively tag and identify novel cancer driver genes in different solid tumors (Figure 1). In this approach, the vector provirus acts as a molecular tag and the analysis of retroviral integration sites (RIS) allows for the rapid identification of dysregulated cancer driver genes. The completion of the human genome project has facilitated the rapid identification of potential cancer driver genes near RIS in mutagenesis screens. This has led to remarkable progress in this emerging screening approach to identify driver genes that are mutated during cancer progression. Replication-incompetent vectors can identify novel molecular mechanisms for tumor initiation and progression and also potential biomarkers or targets for small-molecule therapeutic drugs to improve patient outcomes.

## 2. Identifying Cancer Genes by High-Throughput Sequencing Is Challenging

High-throughput sequencing approaches have identified dysregulated genes associated with cancer progression [27,28,29,30]. The sequencing of matched tumor and normal tissues across different cancer types have identified previously known and unknown cancer genes [31]. In breast cancer, high-throughput sequencing has identified mutations related to tumor clonal evolution, heterogeneity and metastasis [32]. High-throughput sequencing has also identified drug resistance genes. For example, whole-exome sequencing of mutant BRAF^v600^ metastatic melanoma patient tumors identified RAF kinase inhibitor drug resistance genes and the associated mechanisms in melanoma progression [33]. These examples highlight the power of high-throughput sequencing to identify genes that drive tumor growth, metastasis and drug resistance. However, the majority of mutations identified by high-throughput sequencing are passengers that do not provide a clonal advantage to cancer cells during tumor development [34]. Thus, it remains a major challenge to distinguish passenger mutations from driver mutations using this approach [35,36,37].

## 3. Insertional Mutagenesis Screens

### 3.1. Transposon Based Insertional Mutagenesis Screens

The Sleeping Beauty (SB) transposon system is a DNA vector flanked by inverted repeats that is inserted into the genome using a transposase that is provided in trans [38]. SB transposons have been widely used in mutagenesis screens for cancer gene discovery in mouse models [14,15]. The SB system uses a genome-wide “copy and paste” mechanism for transgene delivery, where the transposase excises transposons from their original location and reintegrates them elsewhere in the genome [38]. However, transposons screens in mice and mammalian systems are limited due to the lack of efficient transposition [39]. When delivered exogenously on plasmid DNA, transposons can mutagenize a wide array of tissues [40]. In transposon-based mutagenesis, a library of cells with insertions are created in which genomic loci are dysregulated by the integrated transposon [41]. The genomic loci having recurrent insertions found in multiple independent tumors, a common integration site (CIS), indicates possible selection for a nearby gene that drives cancer progression [13]. However, the transposon approach has several limitations. The use of a “copy and paste” mechanism for transposition can lead to multiple integrants in individual cells which then complicates the identification of tagged driver genes from tagged passenger genes. Transposons also exhibit “local hopping”, which is the reintegration of transposons near its original integration site in the genome, which also complicates the identification of driver genes [15,18]. Together, these properties result in a wide spectrum of secondary mutations that can mask the identification of causal driving mutations in malignant tumors, and make it difficult to isolate drivers from passengers.

There are additional limitations to the SB system. The low transposition frequency of transposons in somatic cells has led to modifications of SB to cause rapid tumor development in mice. However, the generation of knock-in transgenic mouse lines that carry high copy numbers of transposons has some challenges. It takes longer to generate high copy lines and 50%–75% of embryos may die from developmental defects caused by DNA double strand breaks from SB excision [14,15,42]. SB has a high propensity to integrate in AT-rich [18,43] and DNA methylated [44,45] regions as well as in regions with high-mobility group B1 (HMGB1) proteins in mouse cells [46], contradicting earlier reports that transposons integrate randomly in the genome. This might limit their ability to identify cancer genes in regions that are not efficiently targeted by transposons.

The piggyBac (PB) transposon system has recently been used as an alternative to SB in mammalian cells. It has the capacity to deliver larger transgene cassettes than SB, 9.1–10 kb of transgene DNA, and excision of PB is more precise than SB in human cells [39,47]. When PB transposase is fused to a transcription factor, it directs the insertion of PB transposon close to where the transcription factor is bound causing site specific insertional bias [48]. The local hopping of PB is also less severe than SB transposition [49]. Despite these advantages over SB, PB still leads to multiple insertions during transposition and local hopping. Thus, identifying true driver genes from passengers in screens utilizing PB is still challenging.

### 3.2. Insertional Mutagenesis Using Replication-Competent Retroviruses

Replication-competent retroviruses such as Moloney murine leukemia virus (MoMLV) or mouse mammary tumor virus (MMTV) have been used in insertional mutagenesis screens. However, the use of replicating retroviruses limits the screen to cells that are permissive for viral replication. Replicating MoMLV can infect susceptible transgenic mouse strains that develop leukemia and lymphoma malignancies as a result of viral insertions near cancer genes [50,51]. MMTV can infect mouse mammary cells and integrate near cancer genes that promote establishment of mammary tumors [52]. However, MoMLV or MMTV cannot replicate in human cells. Thus, it is not possible to use replicating retroviruses that do not replicate in human cells for screens in human cancer cells and tissues. Further, the multiple viral integrations identified in a single tumor cell from rounds of viral re-infections has made it challenging to use this system to discriminate true causal driver genes from passengers [53].

### 3.3. Replication-Incompetent Vectors Can Be Pseudotyped to Allow Mutagenesis of Essentially Any Mammalian Cell

Replication-incompetent retroviral vectors have been derived from several retroviruses including gammaretroviruses and the HIV-1 lentivirus. These vectors require membrane bound receptors for cellular entry, however the use of an envelope pseudotype allows retroviral vectors to transduce many different cell types. Envelope pseudotyping also can increase vector transduction efficiency and stability. Numerous envelope glycoproteins from other viruses such as amphotropic (AMPHO) murine leukemia virus (MLV), modified feline endogenous virus (RD114), feline leukemia virus type C (FLVC), cocal vesiculovirus and vesicular stomatitis virus (VSV) have been used to generate pseudotyped retroviral vectors [54,55]. VSV glycoprotein (VSV-G) envelope pseudotyping is the most widely used envelope glycoprotein for pseudotyping. Pseudotyped retroviral vectors with VSV-G have broad tropism, and are stable allowing concentration to high titers by centrifugation. Thus, high titer VSV-G pseudotyped γRV (gammaretroviral) and LV (lentiviral) vectors have been used for prostate cancer and breast cancer screens that have utilized human cells in mouse xenotransplant models [21,23,24].

### 3.4. Replication-Incompetent Vectors Can Cause Cancer via Insertional Mutagenesis

**Figure 2:**
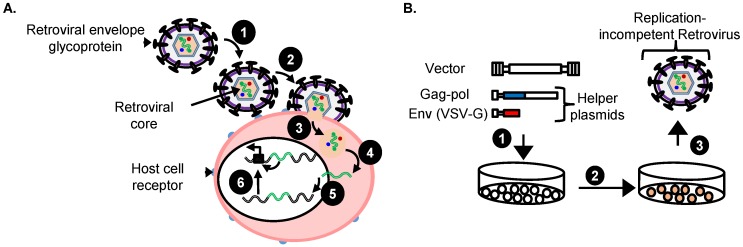
(**A**) Unique retroviral “single hit” during transduction of target cancer cells by replication-incompetent retroviral vector in an insertional mutagenesis screen. Replication-incompetent retroviral vector with a suitable envelope glycoprotein such as VSV-G (1) attach to the target cell receptors causing fusion between the membranes resulting in (2) cell entry of virion core into the cytoplasm where it (3) uncoats and the viral RNA is (4) reverse transcribed into double stranded preintegration DNA and transported into the nucleus where it (5) stably integrates into the chromosome and causes (6) insertional mutagenesis via known mechanisms such as enhancer-activation of a nearby promoter of a proto-oncogene; (**B**) Production of replication-incompetent retroviral vectors. (1) Human embryonic kidney 293 (HEK 293) cells are transiently transfected with vector plasmid and helper plasmids; (2) Vector virions are produced for 72 h; (3) Vector virions are harvested, filtered and concentrated 100-fold by ultracentrifugation for 18 h.

Retroviral vectors are an efficient tool for therapeutic transgene delivery as evidenced by recent successes in hematopoietic stem cell (HSC) gene therapy [56]. During transduction the single stranded RNA genome is reverse transcribed and the viral genome integrates into the host HSC genome (Figure 2).

The integrated retroviral vector provirus is then stably maintained in the host cells as they divide. This allows efficient transmission of a therapeutic transgene from the HSC to all mature blood cells. Diseases successfully treated include X-linked severe combined immunodeficiency (SCID-X1) syndrome [57,58], adenosine deaminase deficiency (ADA) [59,60], X-linked adrenoleukodystrophy [61,62], X-linked chronic granulomatous [63], β-thalassemia [64], and Wiskott-Aldrich syndrome [65]. However, vector-mediated genotoxicity was observed in a SCID-X1 French clinical trial [66]. In this trial, patient HSCs were transduced using a γRV vector that delivered a therapeutic transgene ex vivo and infused back into the patient [57]. Unfortunately, some patients developed leukemia as a result of a γRV vector integration near or in the *LIM* domain only 2 (*LMO2*) oncogene that activated *LMO2* expression [67,68,69]. Other clinical trials have also reported that the retroviral integration(s) near or in proto-oncogenes caused genotoxicity [63,70]. The most common mechanism through which integrated retroviral vectors cause genotoxicity is enhancer-mediated activation of nearby gene promoters [71]. Other known mechanisms through which retroviral vector can cause genotoxicity have previously been discussed [72,73]. These gene therapy studies highlight the power of replication-incompetent vectors to cause cancer, and show that replicating vectors are not required.

## 4. Replication-Incompetent Retroviral Vector Design and Use

### 4.1. Choice of Replication-Incompetent Retroviral Vector Type

Retroviral integration in the genome is a semi-random event influenced by the type of retroviral vector used [74,75,76]. γRV vectors have a high propensity for integrating near expressed gene regions, transcriptional start sites, and promoter regions with CpG islands. They preferentially target the 5′ region of genes [74] and therefore are prone to transactivate a downstream oncogene by enhancer activation. LV vectors integrate preferentially into transcriptionally active gene regions, but less frequently in promoter regions. γRV vectors are more genotoxic than LV vectors, with reported threefold in vitro and tenfold in vivo likelihood of inserting near proto-oncogenes compared to LV vectors in a HSC model [77,78]. Higher LV vector integration loads are needed to match the mutagenic potential of a γRV vector design in a tumor prone mouse model [79]. Further, γRV vectors are often observed near gene classes associated with cell growth even in the absence of strong promoters and may increase the risk of clonal dominance in a cancer mutagenesis screen. γRV vectors have lower titers relative to LV vectors [80] and inefficiently transduce quiescent cells compared to LV vectors as a result of their requirement for mitosis [81,82], thus they have some drawbacks relative to LV vectors. Therefore, the choice of using a LV or γRV vector, can be influenced by the permissiveness of the target cell type for transduction. For quiescent cells that are difficult to transduce LV vectors may be a better choice, but for actively dividing cells γRV vectors may be a better choice as they are more mutagenic.

### 4.2. Replication-Incompetent Retroviral Vector Design

**Figure 3:**
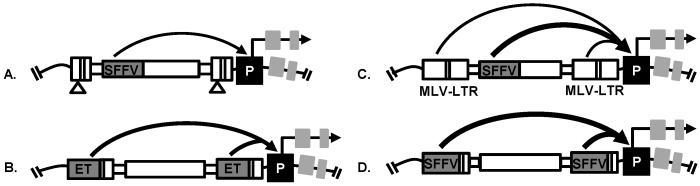
Retroviral vectors used in insertional mutagenesis screens to dysregulate nearby proto-oncogene via known mechanisms including enhancer-mediated activation of gene promoters (P). (**A**) Prostate cancer screen: Self-inactivating LV vector used to identify androgen-independent prostate cancer driver genes; (**B**) Hepatocellular carcinoma screen: LV vector with a hepatocyte-specific promoter (enhanced transthyretin, ET) in the LTR used to identify genes that drive hepatocellular carcinoma progression; (**C**) Breast cancer screen: γRV vector with MLV-derived LTR and with a strong internal spleen focus forming virus (SFFV) promoter used to identify breast cancer metastasis driver genes; (**D**) Drug resistance screen: LV vector with SFFV promoter in the LTR used to identify drug resistance genes in HER 2+ breast cancer and pancreatic adenocarcinoma.

Replication-incompetent retroviral vector design is an important factor affecting viral genotoxicity [78]. Multiple retroviral vector designs have been used for insertional mutagenesis screens with or without transcriptional enhancers in the long terminal repeats (LTR). The transcriptional enhancers in the LTR of vectors have been established as the main activators of oncogenic events that promote progression of cancer. Self-inactivating LV and γRV vectors have the promoter and enhancer elements deleted from the U3 region of their LTR [77,78] and transcription is mediated solely by an internal promoter. These LTR modifications have reduced retroviral vector genotoxicity but self-inactivating vectors are still capable of dysregulating nearby genes when a strong internal promoter is used. For example, a self-inactivating LV vector with a strong internal spleen focus forming virus (SFFV) promoter was capable of dysregulating nearby genes in a prostate cancer mutagenesis screen [23,24] (Figure 3). In addition, a highly genotoxic γRV vector with a MLV-LTR and an internal SFFV promoter was used in a breast cancer mutagenesis screen [21] (Figure 3). The use of either LTR-driven or self-inactivating retroviral vector has proven to be effective in studies performed to identify potential candidate driver genes in different types of cancer, such as prostate cancer [24], hepatocellular carcinoma [22], breast cancer [21,25] and pancreatic adenocarcinoma [25] as we describe later in this review.

### 4.3. Production of Replication-Incompetent Retroviral Vectors

Replication-incompetent retroviral vectors are typically produced by transiently co-transfecting helper and transfer plasmids into the HEK 293 cell line (Figure 2) [83]. The VSV-G envelope pseudotype typically used for replication-incompetent retroviral vectors allows these vectors to be concentrated to high titers by ultracentrifugation without losing their infectivity. The viral vectors and helper plasmids are available from repositories such as Addgene and detailed protocols for vector production are available [55]. This approach of vector production is simple, efficient and reproducible.

### 4.4. Vectors with a Drug Resistance Gene Can Be Used at a Low MOI to Reduce Passenger Mutations

It is important to have most cells mutagenized during a screen to improve the probability of identifying a cancer driver gene. However, if a high MOI is used it may increase the probability of passenger mutations due to multiple integrations in a single cell. Therefore, to improve replication-incompetent retroviral vector efficiency in mutagenesis screens, the vector construct can be modified to contain a suitable drug-selection transgene to eliminate untransduced cells after vector exposure [84]. This avoids the use of a high MOI during transduction. For example, cells transduced at an MOI of 0.3 and 0.9 resulted in detection of one vector copy and nine vector copies per cell, respectively [85,86,87]. Thus, using a low MOI minimizes the undesired effects of passenger mutations resulting from more than one viral insertion in a cell that can complicate identification of drivers [84]. In a breast cancer mutagenesis screen, cells were transduced at a low MOI of 0.2 using an LTR-driven γRV vector with a strong SFFV promoter driving a neomycin drug resistance gene that allowed for G418 selection post-transduction [21] (Figure 3). Since the in vitro culturing of transduced cells can influence the clonal selective pressures [88,89], the length of in vitro culturing of transduced breast cancer cells prior to transplantation into animal host was minimized to 16 days [21]. This approach of transducing cells with a low MOI and then selecting transduced cells avoids passenger mutations, while still ensuring a high percentage of cells used in the screen are mutagenized.

## 5. The Identification of RIS That Tag Cancer Driver Genes

The identification of RIS in cancer cells provides a powerful approach to identify the genomic loci harboring nearby novel genes that drive cancer progression. The identification of CIS is particularly beneficial as it improves the likelihood that a tagged gene is a driver gene. In a retroviral insertional mutagenesis screen, cell clones containing a vector provirus in or near a proto-oncogene or tumor suppressor gene that have a selective advantage will be enriched during tumorigenesis. The mapping of RIS in tumors identifies genes that mediate cancer progression [12]. These RIS are located in or near candidate genes. Typically, to identify RIS, the genomic DNA is obtained from tumors that have developed from transduced cancer cells in an insertional mutagenesis screen. The LTR-chromosomal junctions are then amplified using well-established approaches such as shuttle vector rescue [21,24], linear amplified mediated-PCR (LAM-PCR) [22,25] or high-throughput modified genomic sequencing-PCR (MGS-PCR) [23] as described below. These candidate tagged driver genes can be oncogenes that require only a single retroviral insertion to be activated, or a tumor suppressor gene that requires only a single viral insertion to cause loss of function of one allele resulting in haplo-insufficiency. The tagged driver genes can encode any protein class involved in cancer progression including transcription factors, kinases, phosphates, cytokines, chemokines, chromatin modulators, cell cycle and apoptosis regulators. One limitation to this approach is that the mapping of RIS located in repetitive genomic regions can still be challenging.

### 5.1. Shuttle Vector Rescue Approach

The shuttle vector rescue approach overcomes some limitations of PCR-based approaches for identifying RIS [75]. The retroviral shuttle vector must encode a bacterial drug resistance gene, such as kanamycin. For the shuttle vector rescue approach the genomic DNA is extracted from retroviral mutagenized cancer cells and sheared randomly to obtain DNA fragments. These fragments are end repaired, ligated and transformed by electroporation into bacteria. Plasmids obtained from the kanamycin resistant colonies are high-throughput sequenced with primers specific to the vector 3′ LTR. The integrated provirus LTR-chromosomal junctions are identified and sequence reads can be aligned to the human genome using the BLAST-like alignment tool (BLAT) [90]. The lengths of sequences obtained by shuttle vector rescue are usually longer than those produced by PCR that allows for improved alignment scores, which can improve detection in repetitive regions. The shuttle vector rescue approach has been used for efficient identification of RIS in breast cancer [21] and prostate cancer [24] insertional mutagenesis screens. A γRV shuttle vector approach identified previously known (*WWTR1, RIN1*) and also novel (*SHARPIN)* breast cancer metastasis genes [21]. However, despite these successes shuttle vector rescue approach can lack sensitivity to detect RIS in tumors with low vector copy number [23].

### 5.2. LAM-PCR

The LAM-PCR method has been used to identify RIS in retroviral based insertional mutagenesis screens [12]. The genomic DNA samples derived from retroviral transduced cells are digested with restriction enzymes and amplified with retroviral specific biotinylated LTR primers. The amplified sequences are captured with streptavidin-magnetic beads and isolated. Linker cassettes are ligated onto the genomic end of captured target DNA fragments and exponential-PCR(s) is performed using LTR and linker-specific primers. Nested PCR is performed with LTR and linker-specific primers on the exponentially amplified PCR product. The PCR product is separated, isolated, purified, concentrated and high-throughput sequenced to identify RIS. PCR-based approaches have technical challenges associated with PCR amplification that can limit the efficiency of RIS detection. For example, if a primer site is distantly located from the integration site then the amplification of the targeted region having that integrant is inefficient. However, this approach has identified RIS in forward insertional mutagenesis screens [22,25].

### 5.3. MGS-PCR

A novel high-throughput MGS-PCR based method has been designed recently to overcome some limitations of LAM-PCR and shuttle vector rescue approach [91,92]. MGS-PCR does not rely on restriction digest of genomic DNA and allows analysis of the number of different span or sheared lengths to evaluate clonality. This method has recently been used to identify RIS in a prostate cancer mutagenesis screen [23]. Briefly, the genomic DNA is randomly sheared to obtain fragments and linker cassettes ligated to both ends. Small DNA fragments are excluded that might skew amplification and exponential PCR are conducted using a biotinylated LTR-specific primer and a linker-specific primer. A total of 0.8–1.2 million sequence reads per sample were obtained which shows that MGS-PCR has a much higher sensitivity than the previously described shuttle vector rescue approach [92]. The sequences obtained were analyzed using the vector integration site analysis (VISA) bioinformatics tool to identify vector-chromosome junctions and determine RIS within the human genome (hg38) as well as nearby genes [93].

## 6. Retroviral Insertional Mutagenesis Screens to Identify Cancer Driver Genes

### 6.1. Prostate Cancer

Prostate cancer is the most common diagnosed form of cancer and the second leading cause of cancer related deaths in US men [1]. In the initial stages, prostate cancer is androgen-dependent and androgen-deprivation therapy is usually the standard first line of therapy. At this early stage, the treatment reduces tumor size. However, at later stages, some patients develop androgen-independent prostate cancer that is more aggressive, and metastasis causes mortality. Therefore, it is critical to identify genes that drive the progression of androgen-independent prostate cancer that might be potential drug targets or biomarkers. Schinke et al. [24] used a self-inactivating replication-incompetent LV vector (Figure 3) in a retroviral insertional mutagenesis screen to identify potential driver genes in androgen-independent prostate cancer. This insertional mutagenesis screen was designed to model the progression of androgen-independent prostate cancer using both in vitro and in vivo assays. In this study, androgen-dependent LNCaP cells were transduced using a replication-incompetent self-inactivating LV vector with a strong internal SFFV promoter. Androgen-dependent LNCaP cells were chosen for this screen because they can become androgen-independent by culture in charcoal treated media that is androgen-deficient. This design provided a tractable in vitro system to screen for genes that drive androgen-independent prostate cancer. LNCaP cells mutagenized by self-inactivating LV vectors were cultured in androgen-deprived media to model the progression of androgen-independent prostate cancer in patients. In androgen-independent condition, clones with integrations near genes that confer selective-growth advantage become dominant. These clones potentially contain RIS near androgen-independent prostate cancer driver genes. Analysis of genomic DNA from the in vitro androgen-independent prostate cancer cells revealed 21 unique RIS. In the in vivo screen, mutagenized LNCaP cells were subcutaneously transplanted into male immunodeficient mice and after primary tumor development the animals were castrated to establish an androgen-deficient environment that models what occurs in prostate cancer patients undergoing androgen deprivation therapy. Thus, the primary and metastatic tumors that developed were selected for androgen-independent growth. The provirus integration sites were determined using a the shuttle vector rescue approach [90] as described earlier in this review and 54 RIS were identified. Using this approach, the RIS were identified near a known prostate cancer gene *PTRF* as well as other genes that were not previously implicated in prostate cancer including *ATPAF1, GCOM1, MEX3D,* and *TRPM4* (Table 1).

Nalla et al. [23] used a self-inactivating LV vector (Figure 3) in a insertional mutagenesis screen and identified genes that promote androgen-independent prostate cancer. This study used a high-throughput MGS-PCR to identify RIS in in vivo orthotopic tumors that developed under androgen deficient conditions in castrated mice. One to four million sequence reads were obtained per tumor and the RIS and nearby genes determined using VISA [93]. A total of 394 unique RISs were recovered from tumors. To assess the expression of candidate prostate cancer genes in patients, publicly available prostate cancer data were used to perform a meta-analysis across 16 Oncomine^TM^ datasets and cBioPortal. Eleven candidate prostate cancer genes were identified: *GLYATL1, FLNA, OBSCN, STRA13, WHSC1, ARFGAP3, KDM2A, FAM83H, CLDN7, CNOT6* and *B3GNT9* (Table 1). CIS were identified near the genes *OBSCN, KDM2A* and *ARFGAP3*. All 11 genes that were identified had genetic alterations in prostate cancer patients with *FAM83H* being the most frequently mutated gene. *KDM2A, FAM83H* and *GLYATL1* were validated in vitro using a doxycycline inducible shRNA system. Silencing of *KDM2A, FAM83H* and *GLYATL1* significantly inhibited the clonogenicity of prostate cancer cells in vitro. Using a proliferative recovery assay, the knockdown of *KDM2A* significantly inhibited recovery arrest of prostate cancer cells in androgen depleted conditions. Further, using SurvExpress, the prognostic significance of candidate genes in predicting the clinical outcome of prostate cancer patients after therapy was evaluated. Gene combinations of *OBSCN, FAM83H, CLDN7* and *ARFGAP3* significantly predicted the risk of re-occurrence after treatment, demonstrating that the genes identified through this screen can have high predictive value in patients and thus are potential prostate cancer biomarkers.

Both the in vitro and in vivo prostate cancer screens clearly demonstrated that a retroviral-based insertional mutagenesis can be an efficient tool to identify androgen-independent prostate cancer driver genes. The MGS-PCR approach was found to be more efficient in rescuing RIS in tumorigenic cells than shuttle vector rescue. In future studies it will be interesting to determine if these genes can be used as therapeutic targets or biomarkers that can stratify patients to allow for targeted treatments.

### 6.2. Breast Cancer

**Figure 4:**
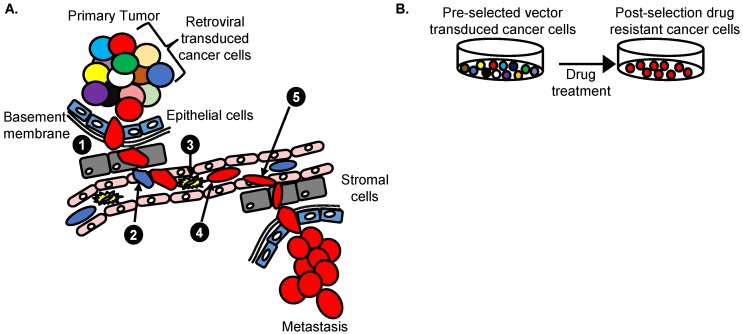
Retroviral insertional mutagenesis screens. (**A**) Approach to identify cancer metastasis driver genes in vivo. Retroviral vector transduced cancer cells are tagged and indicated as different colors. Cells with a selective advantage to (1) invade underlying basement membrane (2) intravasate (3) withstand circulatory pressure and (4) migrate (5) extravasate and metastasize to distal organs (Red) can be identified; (**B**) Approach to identify cancer drug resistance genes in vitro. Treated cancer cells are transduced with retroviral vector leading to a polyclonal population of cells with RIS that are randomly distributed in the genome. Following increased drug dosing, the cells with RIS near genes that confer a selective advantage to survive and grow will be enriched (Red). Analysis of RIS on the red cells identifies candidate cancer drug resistant driver genes that promote drug resistance in cells.

A γRV shuttle vector insertional mutagenesis screen identified *SHARPIN* as a novel breast cancer metastasis driver gene in vivo [21]. Unlike the prostate cancer mutagenesis screen that used a LV vector [24], a highly genotoxic replication-incompetent γRV shuttle vector containing enhancers in the MLV-LTR and in the internal SFFV promoter capable of dysregulating nearby genes was used (Figure 3). This γRV shuttle vector construct also contained a neomycin phosphotransferase transgene cassette that allows for selection of transduced cells in culture. This system allowed for transduction of breast cancer cells at a very low MOI to minimize the occurrence of passenger mutations that might mask driver mutations as described earlier. The selection of transduced cells in vitro prior to in vivo screening was performed to ensure that the library of breast cancer cells used in the screen contained only transduced cells. For in vivo screening, γRV vector transduced breast cancer cells were transplanted orthotopically into the mammary fat pad of immunodeficient mice to model the progression of breast cancer metastasis in patients. This in vivo screening approach has the potential to identify genes that mediate various breast cancer metastasis processes including the ability of cells to; proliferate, invade, undergo epithelial mesenchymal transition, intravasate, withstand the circulatory environment, and extravasate to ultimately colonize secondary sites (Figure 4). Clones that withstand these selective pressures are hypothesized to be enriched in the metastatic tumors. The shuttle vector rescue approach was used to identify RIS in metastatic tumors and analyzed using VISA [93]. Eight unique RIS were identified in or near transcription start sites and CpG islands of genes as expected for the γRV vector.

In this screen, patient microarray data from the publicly available Oncomine™ database (https://www.oncomine.org/) were interrogated to determine the expression levels of candidate breast cancer genes identified by γRV vector insertional mutagenesis. This enables independent validation of candidate driver genes using patient data. Four genes *WWTR1, RIN1, MAF1* and *SHARPIN* were identified having significant expression levels in breast cancer patients by meta-analysis (Table 1). *WWTR1* was previously implicated in breast cancer metastasis and drug resistance [94]. *RIN1* was identified as a breast cancer tumor suppressor gene [95]. *SHARPIN* was involved in prostate cancer [96] and breast cancer [97] progression but its role in breast cancer metastasis was still unknown, while *MAF1* had no known involvement in cancer progression. *SHARPIN*, the top candidate metastasis driver gene was validated in vitro and in vivo using an inducible shRNA system. This system allows for use of isogenic cells where the experimental arm is treated with doxycycline to induce knockdown of targeted genes (Figure 1). SHARPIN knockdown in breast cancer cells significantly reduced clonogenicity in vitro and metastasis in vivo providing independent validation that *SHARPIN* affects breast cancer metastasis. Meta-analysis of Oncomine^TM^ breast cancer patient microarray data showed a significant increase in *SHARPIN* expression in patients and *SHARPIN* expression affected metastasis free survival in breast cancer patients after adjuvant chemotherapy treatment as identified by SurvExpress [98]. These findings illustrate that retroviral insertional mutagenesis screens can efficiently identify genes such as *SHARPIN* that can be used as potential biomarkers or therapeutic drug targets for small drug molecules. The use of two independent sources, the mutagenesis screen and the public microarray data, can identify clinically relevant candidate genes.

### 6.3. Hepatocellular Carcinoma

Hepatocellular carcinoma is the third leading cause of global malignant cancer deaths [99]. High-throughput sequencing has been used to identify some cancer genes that are involved in disease progression but the accumulation of passenger mutations in these screens have made it difficult to identify causal driver mutations [100]. Based on their previous success in inducing mutagenesis using a LV vector, Ranzani et al. [78] performed a retroviral insertional mutagenesis screen using a replication-incompetent LV vector to identify novel hepatocellular carcinoma driver genes in in vivo mouse models [22]. This screening system was adapted specifically to induce hepatocellular carcinoma and allow identification of pathogenic pathways. They initially used a LV vector with a hepatocyte-specific enhanced transthyretin (ET) promoter in the LTR (Figure 3) to induce hepatocellular carcinoma mutagenesis in *Cdkn2^−/−^ Ifnar^−/−^* mice which are sensitive to genotoxic mutations. They then used *Pten* null mice (*Pten^flox/flox^ AlbCre^+^*) to investigate the effects of inflammatory microenvironment in non-alcoholic hepatic adenomas and assess hepatocellular carcinoma progression. Thirdly, a wild-type mice was treated with carbon tetrachloride (CCl_4_) to investigate the development of chronic liver injury without progression to hepatocellular carcinoma. All three mouse models were used to induce hepatocellular carcinoma at higher frequency than the genotype matched controls after intravenous administration of LV vectors. These screens show the flexibility of retroviral insertional mutagenesis screens. In this study, the RIS were retrieved by LAM-PCR as described earlier in this review. Four CIS were identified in multiple independent liver tumors harboring the hepatocellular carcinoma driver genes *Fign*, *Braf*, *Sos1*, *and Dlk1-Dio3.* Reverse-transcriptase PCR (RT-PCR) was performed and retroviral derived fusion transcripts were identified with the truncated proteins products having increased cellular activities. LV vector integrations in *Dlk1-Dio3* region generated fusion transcripts with full length *rtl1.*

The genes identified in the screen were validated in vivo using a retroviral screening approach with a self-inactivating LV vector that contained an internal hepatocyte specific promoter (ET promoter) and Mir142 sequences in the 3′ untranslated region (3′ UTR). Mice treated with self-inactivating LV vectors encoding truncated *SOS1* and *BRAF*, and full-length or truncated *FIGN* developed hepatocellular carcinoma. Gene set enrichment analysis (GSEA) signatures showed that *Braf*, *Fign* and *Rtl1* expression were enriched in hepatocellular carcinoma compared to non-tumorous tissues. These genes were implicated in multiple cancer progression processes such as metabolism, cell cycle, growth and proliferation. The WNT signaling pathway was deregulated in tumors expressing *Fgn.* Further analysis of normal and hepatocellular carcinoma microarray patient data showed *SOS1*, *BRAF* and *FIGN* were significantly upregulated (Table 1). Patients with high expression levels of wild type SOS1 in hepatocellular carcinoma had increased survival. *BRAF*, *FIGN* and *RTL1* gene signatures identified in murine hepatocellular carcinoma had relevance in human liver disease; *BRAF* and *RTL1* were downregulated while *FIGN* was upregulated in patients and provided means to stratify these patients according to disease phenotype, stage and disease-free survival. The genes identified by LV vector mutagenesis were implicated in other types of cancers including glioblastoma, oligodendroglioma, melanoma, testicular teratoma, and ovarian endometriosis. The LV vector insertional mutagenesis screen used in this study proved effective for detecting early cancer driver genes that had clinical relevance in human hepatocellular carcinoma disease progression. The identified genes were able to stratify patients into different hepatocellular carcinoma tumor grades 1, 2 and 3. 

## 7. Retroviral Vector Screens to Identify Cancer Drug Resistance Genes

### 7.1. HER 2+ Breast Cancer

Ranzani et al. [25] utilized a LV vector-based insertional mutagenesis screen to identify genes responsible for drug resistance in HER 2+ breast cancer. Drug resistance has been observed in many cancers [101,102,103] and few genes associated with drug resistance have been identified by high-throughput sequencing [104]. As highlighted earlier in this review, breast cancer patients expressing HER 2+ have been treated using targeted monoclonal antibodies, but the incidence of tumor relapse is still very high [25]. The researchers in this study designed a forward insertional mutagenesis screen using a replication-incompetent self-inactivating LV vector to identify genes involved in resistance of lapatinib, a drug approved for treatment of metastatic HER 2+ breast cancer. In this screen, HER 2+ drug-sensitive BT474 and SKBR3 breast cancer cell lines were transduced with a highly genotoxic LV vector with a strong SFFV promoter in the LTR to generate drug-resistant clones (Figure 3). The cells were transduced at varied MOIs 1, 10 and 100 (SKBR3) and 0.5, 5 and 50 (BT474). The LV vector transduced cells were cultured with media containing different concentration of lapatinib ranging from 0.5–2 µM. Lapatinib induced cell death in breast cancer cells that were drug sensitive. A few LV vector transduced cells developed drug-resistant clones that became dominant (Figure 4). Compared to the untransduced controls the LV vector transduced cell cultures had a significant number of lapatinib drug resistant clones.

The drug-resistance genes were identified using LAM-PCR. Unique RIS were identified in drug-resistant clones from LV vector-transduced BT474 and SKBR3 cells. A total 62 CIS were identified in the cell cultures exposed to drugs. CIS were identified near or in *PIK3CA*, *PIK3CB*, *MAP4K3* and *CADM2* genes (Table 1). The genes identified were dysregulated by well-known insertional mechanisms such as enhancer-mediated activation of gene promoters, and chimeric transcripts generated by cryptic vector splice sites from the vector 5′ LTR. The clinical relevance of drug resistant genes identified in HER 2+ breast cancer was analyzed by a forced expression screen where a retroviral vector designed to express the drug resistance gene in the targeted cells was used to determine the pro-survival effect of cells in lapatinib. They designed a low genotoxic self-inactivating LV vector with an internal SFFV promoter to drive the transcription of potential lapatinib resistance genes (*K111N*-mutant *PIK3CA* or wild type *PIK3CB)* in HER 2+ cells. BT474 cells transduced with the *K111N-*mutant *PIK3CA* and SKBR3 cells transduced with *PIK3CB* had a significant survival advantage upon lapatinib administration compared to the mock-treated cells. Breast cancer patient data were used to assess the clinical relevance of *PIK3CA* and *PIK3CB* in patients*. PIK3CA* was associated with a worse prognosis and drug-resistance for lapatinib and tratuzumab. Increased expression of *PIK3CB* was associated with decreased overall survival, and relapse-free survival in HER 2+ breast cancer patients. Other novel breast cancer lapatinib resistance genes were identified in known cancer pathways such as PI3K and in non-coding RNAs such as *LINC00308, MIR181A1* and *LOC647107* (Table 1).

### 7.2. Pancreatic Adenocarcinoma

In the same study described above [25], using a LV vector screening approach, the researchers designed a drug resistance screen to investigate resistance to erlotinib in pancreatic adenocarcinoma. In this screen, a pancreatic adenocarcinoma cell line was transduced at a MOI of 75 with a LV vector having a strong SFFV promoter in the LTR (Figure 3) and treated with different concentration of erlotinib. LV vector transduced cells resulted in a significant number of drug resistant cells after two weeks in erlotinib treatment. RIS from the drug-resistant clones were retrieved and unique integration sites were identified which harbored CIS in candidate drug resistance genes *SOS1, MROH1* and *LOC100128338.* The integrations were oriented in sense with *SOS1* transcription, suggesting a truncated protein may have caused erlotinib resistance.

## 8. Conclusions

Retroviral insertional mutagenesis is a powerful under-utilized tool for identifying novel cancer driver genes. The identification of cancer driver genes can lead to a better understanding of different processes associated with cancer progression such as metastasis and drug resistance. Retroviral vectors have been used successfully to screen for genes related to prostate [24], breast [21,25], and liver cancer [22]. Their ability to transduce any mammalian cell makes them an ideal tool for many cancer types. Moreover, these insertional mutagens can be engineered to contain tissue specific promoters that can be used to investigate organ-specific diseases [22,105]. Compared to transposons, retroviral mutagenesis screens can be used without the need for animal models specifically tailored to a particular disease phenotype. Retroviral vectors have a propensity to integrate in the 5′ end of expressed genes and have strong enhancer activity [21,22,24] compared to the DNA transposons [13,14], which suggests that combining the two approaches may be advantageous.

The complete annotation of the human genome and the emergence of bioinformatics tools have facilitated retroviral vector insertional mutagenesis screens. Bioinformatics tools such as VISA [93], have the ability to analyze large genomic data sets to identify RIS within minutes in various cancer types [24]. Patient data from publicly available databases such as the Gene Expression Omnibus (GEO), Oncomine^TM^ and cBioPortal can be used to independently corroborate cancer genes discovered using mutagenesis screens (Figure 1). In summary, retroviral vector insertional mutagenesis is a powerful tool to identify novel cancer driver genes for any cancer type, particularly when combined with publicly available patient data.

## Figures and Tables

**Table 1:** Potential cancer driver genes identified by retroviral insertional mutagenesis.

Cancer ^a^	Retroviral Vector ^b^	Screen ^c^	Identified Gene ^d^	Reference ^e^
Androgen-independent prostate cancer	Self-inactivating LV	In vivo	*PTRF, GCOM1, MEX3D*	[24]
		In vitro	*ATPAF1, TRPM4*	[24]
Breast cancer	MLV-LTR γRV	In vivo	*SHARPIN, WWTR1, MAF1, RIN1*	[21]
Androgen-independent prostate cancer	Self-inactivating LV	In vivo	*GLYATL1, FLNA, OBSCN, STRA13, WHSC1, ARFGAP3, KDM2A, FAM83H, CLDN7, CNOT6, B3GNT9*	[23]
Hepatocellular carcinoma	LV-LTR	In vivo	*SOS1, BRAF, FIGN, RTL1*	[22]
HER 2+ breast cancer	Self-inactivating LV	In vitro	*PIK3CA, PIK3CB, MAP4K3, CADM2, SOS1*	[25]
Pancreatic adenocarcinoma	Self-inactivating LV	In vitro	*SOS1, MROH1, LOC100128338*	[25]

^a^ Cancer type where retroviral insertional mutagenesis was used; ^b^ The type of retroviral vector used in the insertional mutagenesis screen; ^c^ The type of insertional mutagenesis screen in vivo or in vitro; ^d^ Candidate cancer genes identified in the mutagenesis screen; ^e^ Referenced study.
